# Genetic Engineering of the Kidney to Permanently Silence MHC Transcripts During *ex vivo* Organ Perfusion

**DOI:** 10.3389/fimmu.2020.00265

**Published:** 2020-02-19

**Authors:** Yuliia Yuzefovych, Emilio Valdivia, Song Rong, Franziska Hack, Tamina Rother, Jessica Schmitz, Jan Hinrich Bräsen, Dirk Wedekind, Cyril Moers, Nadine Wenzel, Faikah Gueler, Rainer Blasczyk, Constanca Figueiredo

**Affiliations:** ^1^Hannover Medical School, Institute of Transfusion Medicine and Transplant Engineering, Hanover, Germany; ^2^Department of Nephrology, Hannover Medical School, Hanover, Germany; ^3^Hannover Medical School, Institute for Pathology, Hanover, Germany; ^4^Hannover Medical School, Institute for Laboratory Animal Science, Hanover, Germany; ^5^Department of Surgery-Organ Donation and Transplantation, University Medical Center Groningen, University of Groningen, Groningen, Netherlands

**Keywords:** transplantation - kidney, organ engineering, HLA, gene therapy, lentiviral vector, organ perfusion

## Abstract

Organ gene therapy represents a promising tool to correct diseases or improve graft survival after transplantation. Polymorphic variation of the major histocompatibility complex (MHC) antigens remains a major obstacle to long-term graft survival after transplantation. Previously, we demonstrated that MHC-silenced cells are protected against allogeneic immune responses. We also showed the feasibility to silence MHC in the lung. Here, we aimed at the genetic engineering of the kidney toward permanent silencing of MHC antigens in a rat model. We constructed a sub-normothermic *ex vivo* perfusion system to deliver lentiviral vectors encoding shRNAs targeting β2-microglobulin and the class II transactivator to the kidney. In addition, the vector contained the sequence for a secreted nanoluciferase. After kidney transplantation (ktx), we detected bioluminescence in the plasma and urine of recipients of an engineered kidney during the 6 weeks of post-transplant monitoring, indicating a stable transgene expression. Remarkably, transcript levels of β2-microglobulin and the class II transactivator were decreased by 70% in kidneys expressing specific shRNAs. Kidney genetic modification did not cause additional cell death compared to control kidneys after machine perfusion. Nevertheless, cytokine secretion signatures were altered during perfusion with lentiviral vectors as revealed by an increase in the secretion of IL-10, MIP-1α, MIP-2, IP-10, and EGF and a decrease in the levels of IL-12, IL-17, MCP-1, and IFN-γ. Biodistribution assays indicate that the localization of the vector was restricted to the graft. This study shows the potential to generate immunologically invisible kidneys showing great promise to support graft survival after transplantation and may contribute to reduce the burden of immunosuppression.

## Introduction

Kidney transplantation remains the best treatment for end stage renal diseases. However, the limited availability of organ donors contribute to increased waiting times and high morbidity and mortality on the waiting list. In Germany there are currently 7,526 patients waiting on a kidney transplantation with waiting times exceeding 10 years (DSO 2018) (1). Despite the advances in histocompatibility and transplant immunology, the success of kidney transplantation relies on the use of powerful immunosuppressive agents that predispose the transplanted patients to infections and malignancies (2, 3). Furthermore, still a considerable number of patients develop graft failure (4–6). The endothelium supports the renal vasculature and modulates crucial mechanisms such as inflammation or thrombosis contributing to the appropriate organ function. The impairment of the endothelium or the mesenchymal transition of the endothelial cells supporting fibrosis is a major cause for acute or chronic allograft rejection. After transplantation, the endothelium represents the frontier between the graft and recipient, thereby being the most important immune checkpoint (7–9). The discrepancies at the human leukocyte antigen (HLA) loci between donors and recipients remain the major cause for antibody medicated rejection (10). HLA expression on the renal endothelium serves as a strong antigenic stimulus and is simultaneously a main driver and a target of allogeneic immune responses (11). Allograft loss after kidney transplantation is the result of a tight and synergistic interplay between innate and adaptive immune responses. This involves complex molecular mechanisms based on T and B-cell activation, autophagy, apoptosis, and inflammatory responses (12). Interaction of HLA with T-cell receptor activates T-cell immune responses that may directly target the allograft or induce the *de novo* formation of donor specific antibodies (DSA). Remarkably, approximately 63% of late kidney allograft dysfunction is a consequence of antibody mediated rejection (ABMR) (13). Donor specific antibodies (DSA) targeting HLA class I antigens support inflammation and induce proliferation. Also, DSA specific for HLA class II are often correlated to chronic allograft rejection and may play an important role in necrosis of endothelial cells (14, 15). Recently, *ex vivo* normothermic organ perfusion has emerged as a promising biotechnological platform to preserve and assess organ quality. An increasing number of studies also suggests the potential of normothermic perfusion to improve quality, resuscitate, and eventually repair the organ (16). Organ gene therapy offers the possibility to modulate intragraft gene signatures involved in renal pathologies or graft survival. Nevertheless, *in vivo* non-viral or viral gene therapeutic approaches have shown so far very low efficiencies and lack of organ specificity. The use of lentiviral vectors allows a permanent genetic modification of cells and tissues, but beside difficulties in their large-scale production and purification so far representing an obstacle to the genetic modification of large solid vascularized organs, *in vivo* approaches lack specificity and are prone to off-target effects (17). Therefore, viral vector-mediated transduction during *ex vivo* organ perfusion may offer a promising approach to generate stable genetically engineered organs (18). Previously, we have demonstrated that silencing HLA expression reduce the strength of allogeneic immune responses *in vitro* and *in vivo* (19–22). Hence, in this study we aimed to induce a stable genetic modification of the kidney during *ex vivo* organ perfusion. In particular, in the interest of a precise regulation, we used RNA interference to downregulate MHC class I and II transcript in a rat kidney transplant model as a strategy to reduce the immunogenicity of the allograft. This could offer many new opportunities in transplant settings and contribute to gender and diversity equality.

## Materials and Methods

### Lentiviral Vector Constructs and Vector Production

The pRRL.PPT.eFS.pre lentiviral vector plasmid encoding for the sequence of a secreted form of luciferase from Oplophorus gracilirostris (NanoLuc, NL) as a reporter gene was used for cloning of an RNAi cassette (18). The cassette consisted of U6 and H1 promoter sequences regulating expression of shRNAs targeting rat beta2-microglobulin (β2m; shβ2m: 5′-GGAAAGAAGATACCAAATA-3′) and rat class II transactivator (CIITA; shCIITA: 5′-GGATATGGAAATGGATGAAGA-3′), respectively. Thus, the ultimate construct was designed to silence rat MHC I and rat MHC II genes expression. A vector containing a sequence for a non-sense shRNA (shNS) was used as a control. Lentiviral vector particles were produced in HEK293T cells cultured in HYPERFlask Cell Culture Vessels (Corning, New York, USA). The cells were grown in Dulbecco's modified Eagle's medium supplemented with 10% fetal calf serum (FCS), 1% glutamine and 2% penicillin-streptomycin until a confluence of 80–90% and then transfected. For the transfection, the shRNA-encoding vector, as well as psPAX2 and pMD2.G plasmids were mixed with polyethylenimine (Polysciences, Warrington, PA, USA). Afterwards, this mix was applied onto the HEK293T cells and incubated for 64 h. Then, the cell culture supernatants were collected and centrifuged at 20,000 g, 16°C for 3 h. Pellets of viral vector particles were resuspended in Williams' Media E (WME) (Thermo Fisher Scientific, Waltham, MA, USA), divided in 1 ml aliquots and stored at −80°C. Viral vector titration was performed by p24 enzyme-linked immunosorbent assay (Cell Biolabs, San Diego, CA, USA).

### Transduction of Rat Kidneys With Lentiviral Vectors During *ex vivo* Perfusion

Rat kidneys were perfused in a system designed to allow for the constant roller-pump driven warm oxygenated perfusion (refer to Supplementary Material for the perfusion system details). Kidneys were perfused with WME media supplemented with 5% BSA, 0.007 M creatinine and 30 mM HEPES, as well as 500 μg/ml Cefazoline, as previously described (23). After 10 min of cold storage upon retrieval, the organs were connected to the perfusion system via a fragment of aorta and renal artery and allowed to gradually rewarm to 26–29°C, while being perfused with gradually increasing pump speed for another 15 min. At this stage the flow rate typically reached 3–5 ml/min and the pressure increased to 30–45 mmHg. Then, 0.8 mg protamine sulfate (Sigma–Aldrich, St. Louis, USA) and 1.5 × 10^11^ shβ2m- and shCIITA-encoding or shNS-encoding vector particles were injected into the system. Afterwards, the kidneys were perfused at further increasing speed and temperature for about 20–25 min until the parameters were stabilized at 80–95 mmHg and 31–32°C, respectively. The entire perfusion time with the viral vector was 2 h. Afterwards, the organs were perfused for 15 min with WME-based perfusion solution containing EDTA-treated blood, followed by another 10 min of washing with WME-based perfusion solution only. During the last two post-vector perfusion stages, the kidneys were cooled down to 12–13°C and placed on ice for transportation to the operating room for transplantation.

### Experimental Animals

All animal experiments were conducted according to the German Animal Welfare law and approved by the local authority (Niedersächsisches Landesamt für Verbraucherschutz und Lebensmittelsicherheit, Oldenburg, Germany). The animals were bred in house and provided by Institute of Laboratory Animals of Hannover Medical School, Hannover, Germany.

Left kidneys were retrieved from Lew.1W(WP)/HanZtm rats and transplanted to Lew/NHanZtm in an orthotropic allotransplantation setting (Supplementary Material contains surgery details). Donors and recipients were 8–9 weeks old males. A group of donor kidneys (*n* = 5) was transduced with the shNS lentiviral vector as a control and another group was transduced with the shβ2m and shCIITA-encoding vector (*n* = 7) during the *ex vivo* perfusion prior to transplantation. Blood samples were collected from the recipient rats before transplantation and weekly thereafter by puncturing the retrobulbar venous plexus with EDTA-coated microtubes (Sarstedt, Nuembrecht, Germany). Plasma was stored at −80°C until needed. In addition, urine collection for 6 h during daytime in metabolic cages was done prior and after ktx in weekly intervals. The urine was stored at −80°C until analysis. A follow up duration was 6 weeks. Afterwards, the recipients were sacrificed in deep general anesthesia and several organs were retrieved. Brain, lung, heart, liver, left native recipient kidney, transplanted kidney, spleen, intestine and bone marrow tissue samples were collected, frozen in liquid nitrogen or preserved in RNA later (Sigma, St. Louis, MO, USA) and stored at −80°C after sacrification. The middle part of kidneys was fixed in 3.5–3.7% pH-neutral buffered formaldehyde (Otto Fischar, Saarbruecken, Germany) and embedded in paraffin for further histological analysis.

### Detection of Secreted NanoLuc Luciferase Reporter Gene Expression

Levels of secreted NanoLuc Luciferase reporter gene in plasma and urine samples were measured with help of Nano-Glo Luciferase Assay System (Promega, Madison, USA) according to the manufacturer's protocol. Briefly, 5 μl of plasma or urine samples was diluted 1:10 with phosphate-buffered saline and an equal volume of Nano-Glo Luciferase Assay Reagent was added. The bioluminescence signal, generated as a result of NanoLuc Luciferase interaction with its substrate furimazine, was measured with a luminometer (Berthold Technologies, Zug, Switzerland) after 3 min of incubation.

### Analysis of the Cytokine Secretion Profile in the Course of Kidney Perfusion

Cytokine levels of rat IL-1α, MIP-1α, IL-6, EGF, IL-10, IL-12p70, IFN-γ, IL-17, IL-18, MCP-1, IP-10, MIP-2, TNF-α, and RANTES were measured in the perfusate samples using magnetic multiplex bead technology and serum matrices (Merck Millipore, Schwalbach, Germany). The perfusate samples collected at 5, 30 min, 1 and 2 h time points after starting the perfusion were centrifuged at 1,500 rpm for 5 min at room temperature and stored at −80°C until the cytokines analysis was performed. The samples were incubated with the beads according to the manufacturer's instructions. The beads were acquired using a Luminex 100/200 device (Luminex Corp., Austin, TX, USA) and the cytokine concentrations were calculated using the Xponent software version 3.1 (Luminex Corp.).

### Real-Time Polymerase Chain Reaction

Renal tissue samples stored in RNA later were used for total RNA isolation with RNeasy Mini Kit (Qiagen, Hilden, Germany) followed by reverse transcription using High-Capacity cDNA Reverse Transcription kit (Applied Biosystems, Foster City, USA). Quantitative real-time polymerase chain reaction (qRT-PCR) was utilized to characterize β2m (Rn00560865_m1; Thermo Fisher Scientific, Waltham, MA, USA) and CIITA (Rn01424725_ m1; Thermo Fisher Scientific) transcript levels in amplification reaction with TaqMan Gene Expression Master Mix (Thermo Fisher Scientific). GAPDH was chosen as endogenous control for normalization (Rn01775763_g1; Thermo Fisher Scientific). All samples were measured in triplicate with StepOnePlus Real-Time PCR System and data processed with StepOnePlus Software v2.3 (Applied Biosystems).

### Lactate Dehydrogenase Activity in Kidney Perfusion Solution

Perfusate samples collected at 5, 30, 60, 90, and 120 min time points in the course of kidney perfusion were centrifuged as described before and stored at −80°C until lactate dehydrogenase (LDH) activity was measured with Cytotoxicity Detection Kit (LDH) (Roche, Basel, Switzerland) according to the manufacturer's instructions. Optical density units of the colorimetric reaction of iodonitrotetrazolium conversion into a red colored formazan were used for comparing LDH release at different time points during kidney perfusions.

### Histology

Formaldehyde-fixed renal tissue samples were embedded in paraffin for cutting. Five micrometer sections were prepared and stained with haematoxylin and eosin (H&E). Pathological evaluation of all relevant renal structures including glomeruli, tubuli, blood vessels, and interstitial tissue was performed. A special attention was given to characteristic features of acute tubular injury (ATI) such as tubular swelling, edema and distension, brush border loss, tubular epithelial lucency, flattening, pyknosis, nuclei loss, luminal debris, and tubular necrosis (epithelial cell death).

### Lentiviral Vector Biodistribution Assay

Six weeks after transplantation, the animals were sacrificed and tissue samples of brain, lung, heart, liver, native kidney, transplanted kidney, spleen, intestine, and bone marrow were collected and frozen in liquid nitrogen as described before. These samples were used for genomic DNA (gDNA) isolation with NucleoSpin Tissue Kit (Macherey-Nagel, Düren, Germany). Isolated gDNA was used in a polymerase chain reaction (PCR) to amplify a 296 bp fragment of the genome-integrated lentiviral vector sequence. BIO-X-ACT Short Mix (Bioline, London, UK) and the primers 5′-AATTCGGTTAAGGCCAGGGG-3′; 5′-GCTGTGCGGTGGTCTTACTT-3′ were used for amplification. The PCR product was then separated by electrophoresis next to Quick-Load Purple 100 bp DNA Ladder (New England Biolabs, Ipswich, USA) on a 2% agarose gel with GelStar Nucleic Acid Gel Stain (Lonza, Basel, Switzerland). Images were captured with ChemiDoc MP Imaging System (BioRad, Hercules, CA, USA) and the band intensities were calculated by densitometric analysis using the BioRad Image Lab 6.0.1 software.

### Statistical Analyses

Data are presented as mean ± standard deviations (SD). For comparison of two groups the Student's *t*-test was used. Comparison of multiple groups with two independent variables was performed by two-way-ANOVA. *p* < 0.05 were considered significant. Statistical analyses were performed using GraphPad Prism v5.0 (GraphPad Software Inc., San Diego, CA, USA).

## Results

### Lentiviral Vector Mediated Transgene Delivery Into Rat Kidneys During *ex vivo* Sub-normothermic Perfusion and Subsequent Transplantation

**Figure 1:**
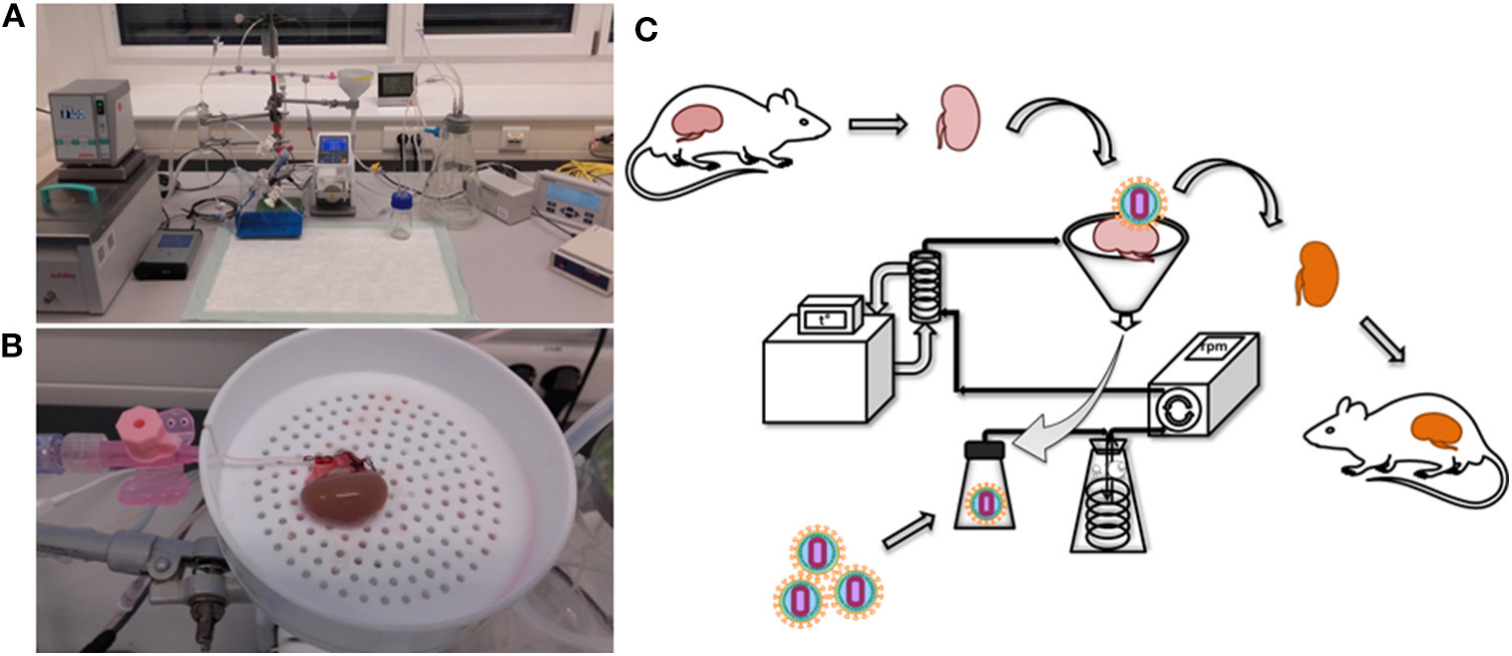
Rat kidney perfusion system for *ex vivo* perfusion and organ genetic engineering. The photographs show the EVKP system **(A)** and the rat kidney connected to the perfusion system via cannulation of the renal artery **(B)**. Schematic representation of the experimental design of the study, including kidney explantation, genetic modification with lentiviral vectors and transplantation **(C)**. Retrieved kidney was placed in the organ container and the renal artery was cannulated and connected to the system. Perfusate flowed from the renal vein into a reservoir for recirculation. Vector particles were injected into the reservoir. A peristaltic pump induced the circulation of the perfusate from the reservoir toward the kidney passing by an oxygenator and glass heat-exchanger. The oxygenated and warmed perfusate entered the kidney via renal arterial cannula.

*Ex vivo* kidney perfusion (EVKP) creates a unique opportunity to genetically engineer the organ. Here, we combine EVKP with lentiviral transduction strategies to genetically modify a rat kidney. For this purpose, we constructed a perfusion system (Figure 1A) to accommodate a rat kidney and allow *ex vivo* perfusion with warm oxygenated WME-based perfusion solution (Figure 1B) and monitoring of major perfusion parameters. In this miniature EVKP system, flow rates of 9–12 ml/min and pressure of 80–95 mmHg under sub-normothermic conditions (32°C) were achieved. Saturation of O_2_ (sO_2_) in the perfusion solution of 65–70% was achieved using a silicone tubing oxygenator submerged in a Büchner flask supplied with carbogen (Supplementary Figure 1). In addition, the perfusion circuit enabled the injection, transport and delivery of lentiviral particles into the rat kidney. Transduced kidneys were subsequently transplanted into allogenic recipients. Experimental design of the study is depicted in Figure 1C and summarized as follows: (1) kidney retrieval from the donor; (2) genetic modification of the kidney with the lentiviral vector during 2 h of EVKP; (3) kidney transplantation (ktx) into the recipient; (4) six weeks monitoring of the transgene expression in the recipient after ktx.

### Detection of the Transgene Expression Post-transplantation

#### NanoLuc Luciferase Reporter Gene Expression

**Figure 2:**
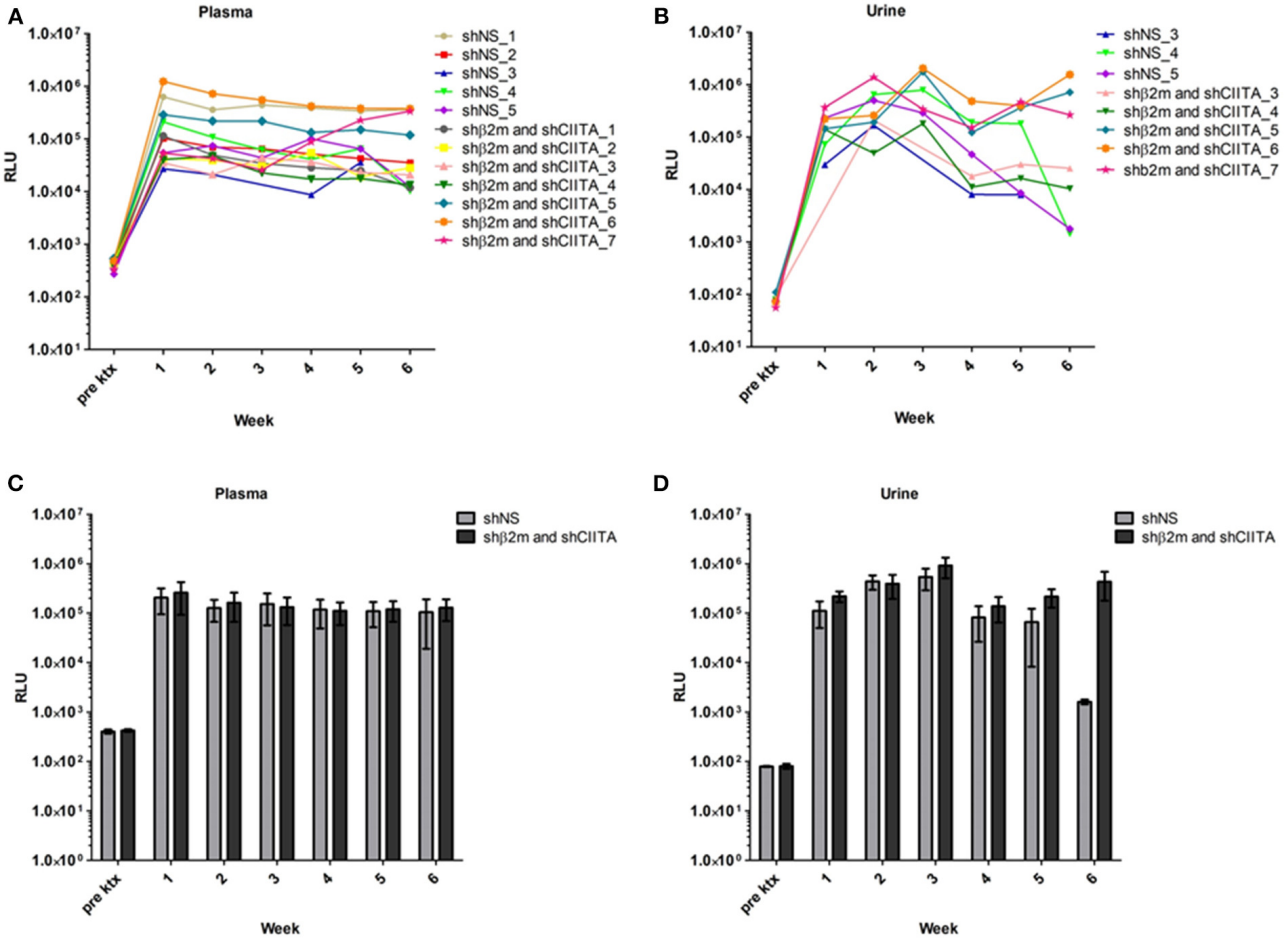
Genetic modification of the kidney during *ex vivo* perfusion. Bioluminescence detected in plasma of the rats transplanted with shβ2m and shCIITA or shNS genetically engineered kidneys. The graphs depict relative luminescence units (RLU) of the secreted NanoLuc Luciferase (NL) reporter gene activity before transplantation and in the course of 6 weeks post-transplantation monitoring **(A)**. Urine bioluminescence levels of the animals transplanted with shβ2m and shCIITA or shNS genetically engineered kidneys. Pre-transplantation NL reporter gene activity levels and NL activity values during 6 weeks after the surgery are shown **(B)**. Mean of RLU detected in plasma of the rats transplanted with shβ2m and shCIITA (*n* = 7) or shNS-expressing (*n* = 5) kidneys (Mean ± SD) **(C)**. Mean of RLU measured in urine of animals transplanted with shβ2m and shCIITA (*n* = 5) or shNS-expressing (*n* = 3) kidneys (Mean ± SD) **(D)**. Pre ktx—pre-transplantation. Statistical analysis was performed by two-way ANOVA. No statistical significance was observed between shNS and the MHC-silence groups.

The lentiviral vector constructs encoding for shNS or shβ2m and shCIITA sequences used in this study also contained the sequence for secreted NanoLuc Luciferase (NL) as a reporter gene. Kidney transduction was measured by evaluation of bioluminescence activity in the body fluids of animals transplanted with genetically engineered kidneys. In comparison to levels of bioluminescence detected in the pre-transplant plasma samples, all animals transplanted with a graft perfused with lentiviral vectors encoding for NL showed an increase in relative luminescence units (RLU) already 1 week post-transplantation 2.67 × 10^4^ to 1.24 × 10^6^ RLU above the pre-transplantation baseline levels. Bioluminescence in plasma samples was detectable during the entire monitoring period and showed 9.64 × 10^3^ to 3.76 × 10^5^ RLU at week 6 (Figure 2A). In addition, weekly urine samples were collected from 8 transplanted rats (3 shNS and 5 shβ2m and shCIITA) (Figure 2B). In urine, bioluminescence increased 1 week post-transplantation, similar to plasma, but reached their peak at week 3 showing 2.06 × 10^6^ RLU above the pre-transplantation baseline. Although, the bioluminescence activity was detectable in urine samples during the entire monitoring time the RLU decreased toward 6 weeks and varied between 1.35 × 10^3^ and 1.56 × 10^6^ (Figure 2B) at the end of the observation time. Means of NL bioluminescence detected in urine and plasma of animals transplanted with shβ2m and shCIITA or shNS renal grafts are shown in Figures 2C,D.

#### Gene Expression Regulation by shβ2m and shCIITA-Encoding Lentiviral Vector

**Figure 3:**
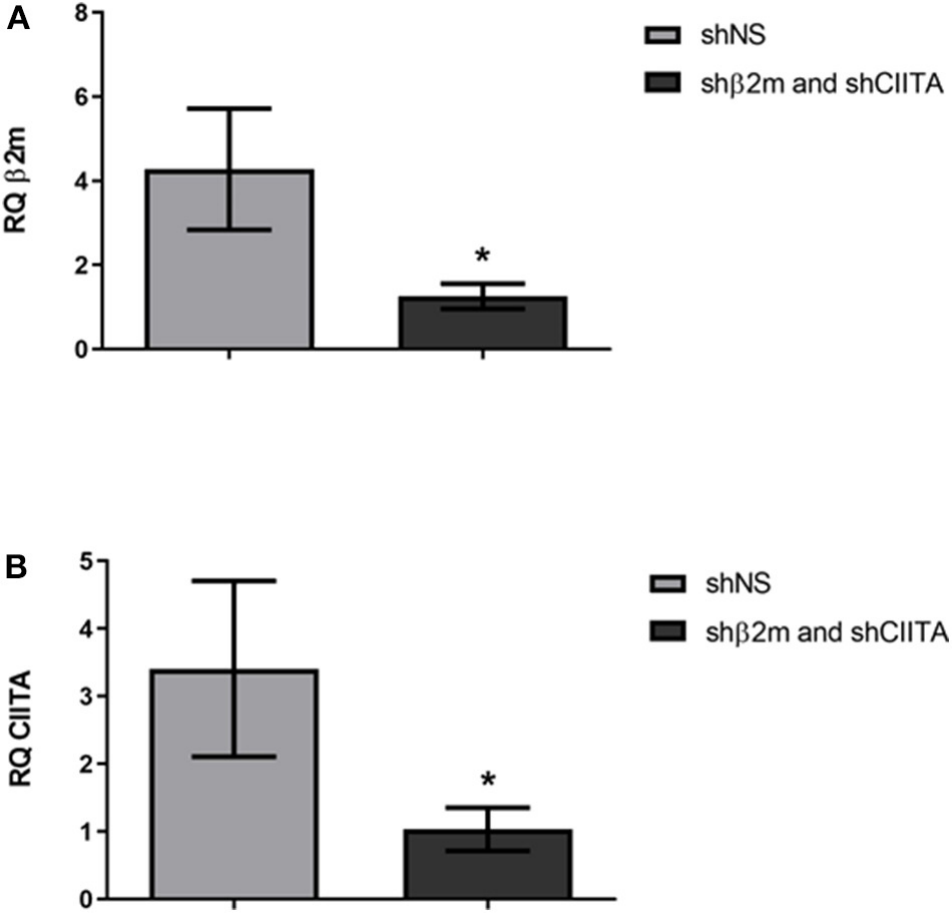
Silencing of β2m and CIITA transcript levels. Relative quantification (RQ) of the β2m gene expression in the kidneys genetically modified with the lentiviral vectors encoding shβ2m and shCIITA (*n* = 7) or shNS (*n* = 5) detected by qRT-PCR (Mean ± SD) **(A)**. RQ values of the CIITA gene transcripts detected by qRT-PCR in shβ2m and shCIITA (*n* = 7) or shNS-transduced (*n* = 5) kidneys (Mean ± SD) **(B)**. Levels of β2m and CIITA gene expression were normalized to GAPDH as housekeeping gene. *p < 0.05 (*t*-test).

Increase in NL bioluminescence levels in plasma and urine are indicators of a successful transduction of the renal tissue during EVKP. Hence, MHC class I and II-related transcript levels were evaluated after 6 weeks post-transplantation with shNS- or shβ2m and shCIITA-expressing kidneys. A downregulation of up to 71% in β2-microglobulin levels was detectable in the kidneys engineered for the expression of shβ2m in comparison to shNS-expressing grafts (Figure 3A). Similarly, rat CIITA transcript levels were decreased by 70% in the kidneys perfused with shCIITA-encoding vector particles in comparison to shNS-treated control kidneys (Figure 3B). These data indicate that the lentiviral vector harboring shβ2m and shCIITA and applied during EVKP has the potential to simultaneously downregulate the expression of MHC class I and II-related transcript levels.

### Assessment of the Kidney Tissue Quality and Integrity in the Course of Sub-Normothermic *ex vivo* Perfusion With Lentiviral Vectors

#### Lactate Dehydrogenase Activity and Histological Analysis

**Figure 4:**
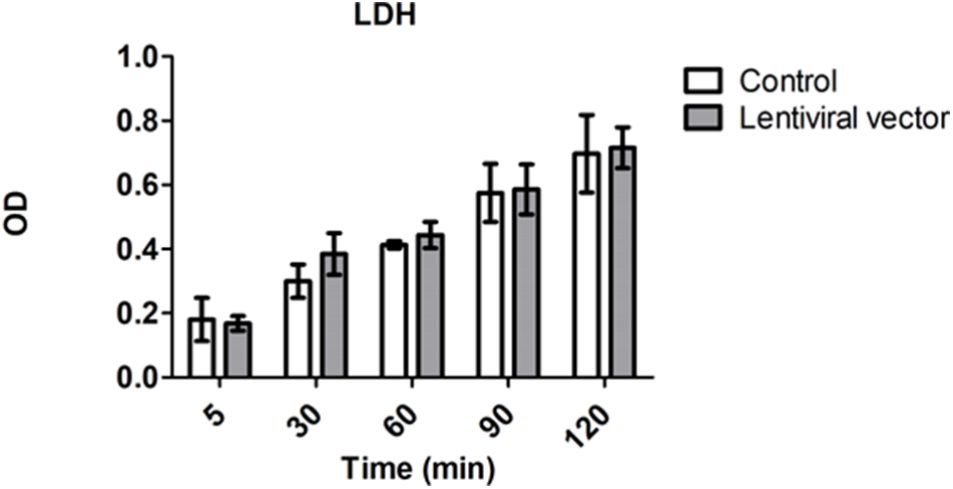
Genetic engineering of the kidney does not cause cell damage. Levels of lactate dehydrogenase (LDH) activity in perfusion solution of the control kidneys (*n* = 3) vs. kidneys subjected to lentiviral vector transduction (*n* = 4) were measured at the beginning (5 min) and every 30 min during EVKP (Mean ± SD). No significant difference in LDH activity was observed between control and lentiviral vector groups as calculated by two-way ANOVA.

**Figure 5:**
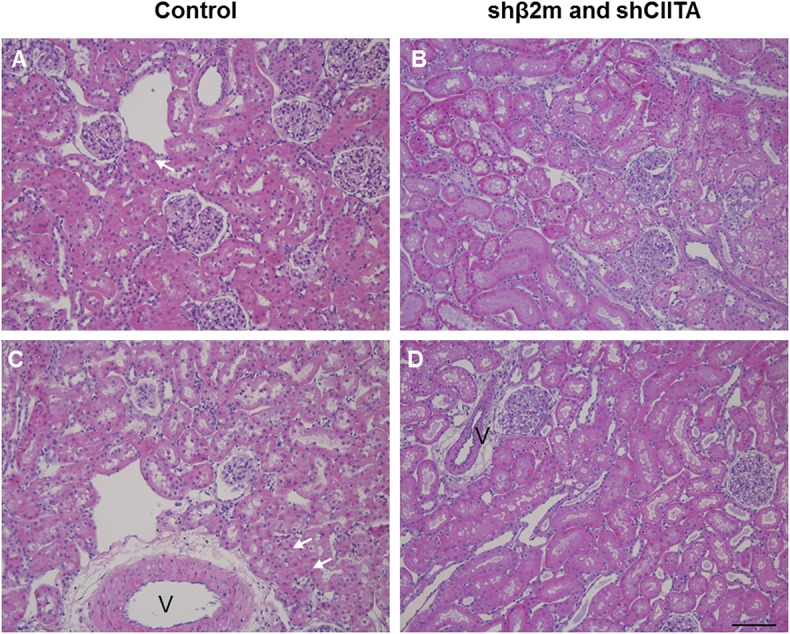
Representative images of perfused kidneys, H&E stain. Perfused control kidneys **(A,C)** or kidneys perfused with lentiviral vector **(B,D)** showed mild to moderate acute tubular injury characterized by tubular vacuolization (arrows, v, vessel). Overall renal morphology was intact in both groups. Images represent individual kidneys from 4 different rats (bar: 100 μm).

Levels of lactate dehydrogenase (LDH) have been used as a marker for tissue integrity (24). In order to estimate the level of potential tissue damage induced by the presence of lentiviral vector particles in the perfusion solution during EVKP, we selected LDH as a tissue damage marker and measured its activity in kidney perfusates. Levels of LDH activity increased with time during EVKP. But importantly, no significant differences in the perfusate LDH levels were observed between kidneys perfused with lentiviral particles and control kidneys perfused only with medium at 5, 30, 60, 90, and 120 min time point (Figure 4). Histopathological findings of the renal tissue samples exposed to the lentiviral vector encoding for shβ2m and shCIITA during EVKP and control kidney samples perfused only with medium were comparable. Both showed potentially reversible mild to moderate acute tubular injury with overall intact renal morphology. No vector-specific damage in the kidneys perfused with the lentiviral vector was detected (Figure 5). These data suggest that application of lentiviral vectors for *ex vivo* kidney genetic engineering under conditions of sub-normothermic perfusion does not cause additional tissue damage in comparison to kidneys perfused without vector.

#### Cytokines Secretion Profile

**Figure 6:**
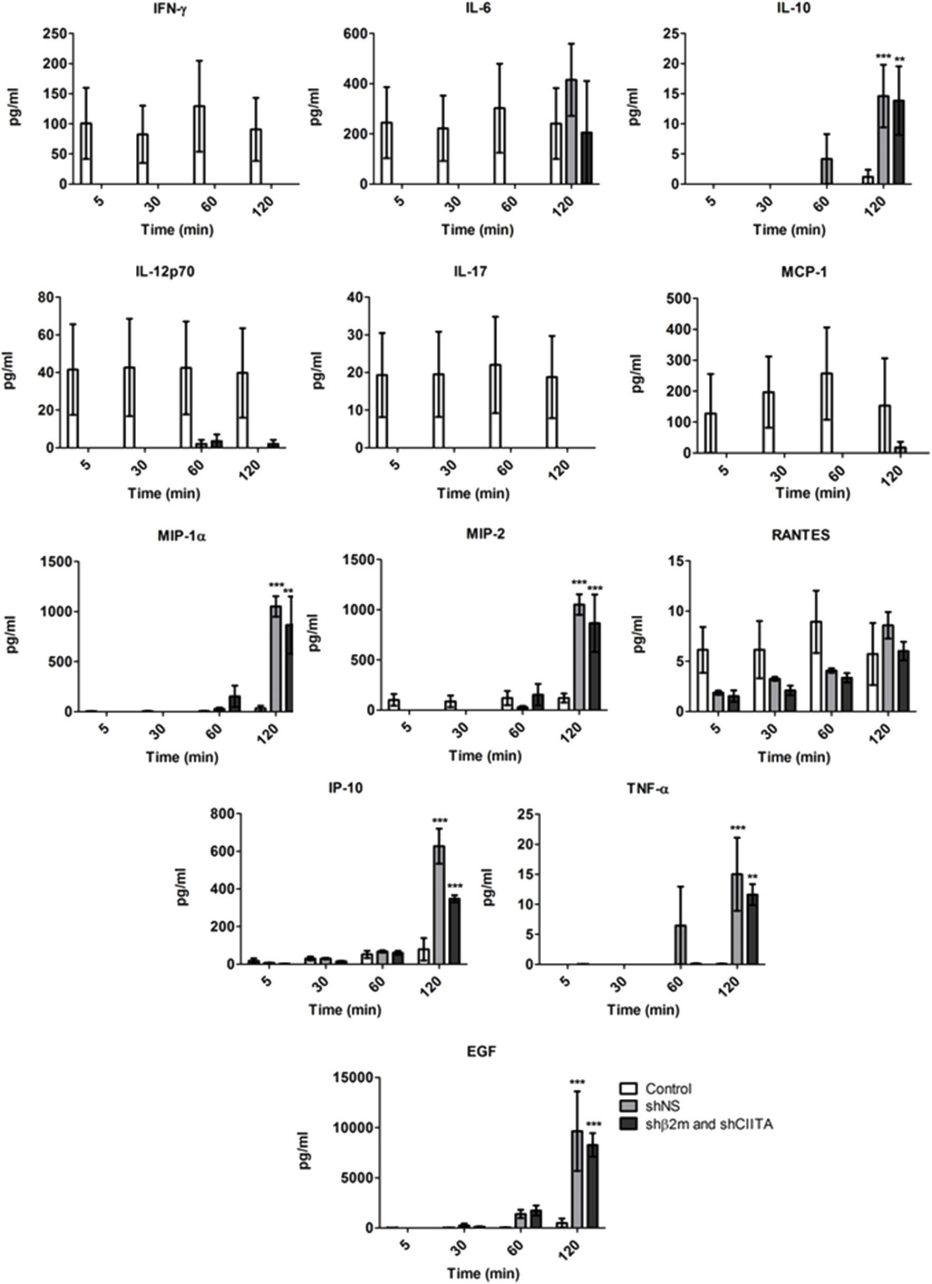
Cytokine secretion signatures during *ex vivo* perfusion and kidney genetic engineering. Cytokine secretion profiles detected in the perfusion solution of the control kidneys (*n* = 4) and kidneys exposed to the shβ2m and shCIITA (*n* = 3) or shNS-encoding (*n* = 3) lentiviral vectors during EVKP (Mean ± SD). ***p < 0.001, **p < 0.01 (shβ2m and shCIITA or shNS vs. control, two-way ANOVA).

Cytokines are important immunomodulatory agents during immune responses after transplantation (25). Therefore, we have characterized potential alterations in the kidney cytokine secretion profile during EVKP in presence or absence of lentiviral vector particles. In comparison to kidneys perfused without vectors, no IL-17 or IFN-γ and lower concentrations of IL-12 and MCP-1 were detectable in kidneys perfused with vector particles during the entire perfusion time. Levels of IL-6 were lower during early perfusion time with lentiviral vectors, but increased at later time point (2 h) to similar concentrations as detected in the kidneys perfused without vectors. In contrast, the secreted levels of IL-10, MIP-1α, MIP-2, IP-10, TNF-α, and EGF were significantly increased in perfusates of kidneys exposed to the lentiviral particles, but only at later time point (2 h). No differences were observed in the secretion patterns of RANTES in kidneys perfused with or without lentiviral vectors. IL-1α and IL-18 were not detected in any of the samples at any time point (Figure 6). These data suggest that perfusion with lentiviral vectors induce an alteration in the pattern of secretion of cytokines.

### Biodistribution Assay

**Figure 7:**
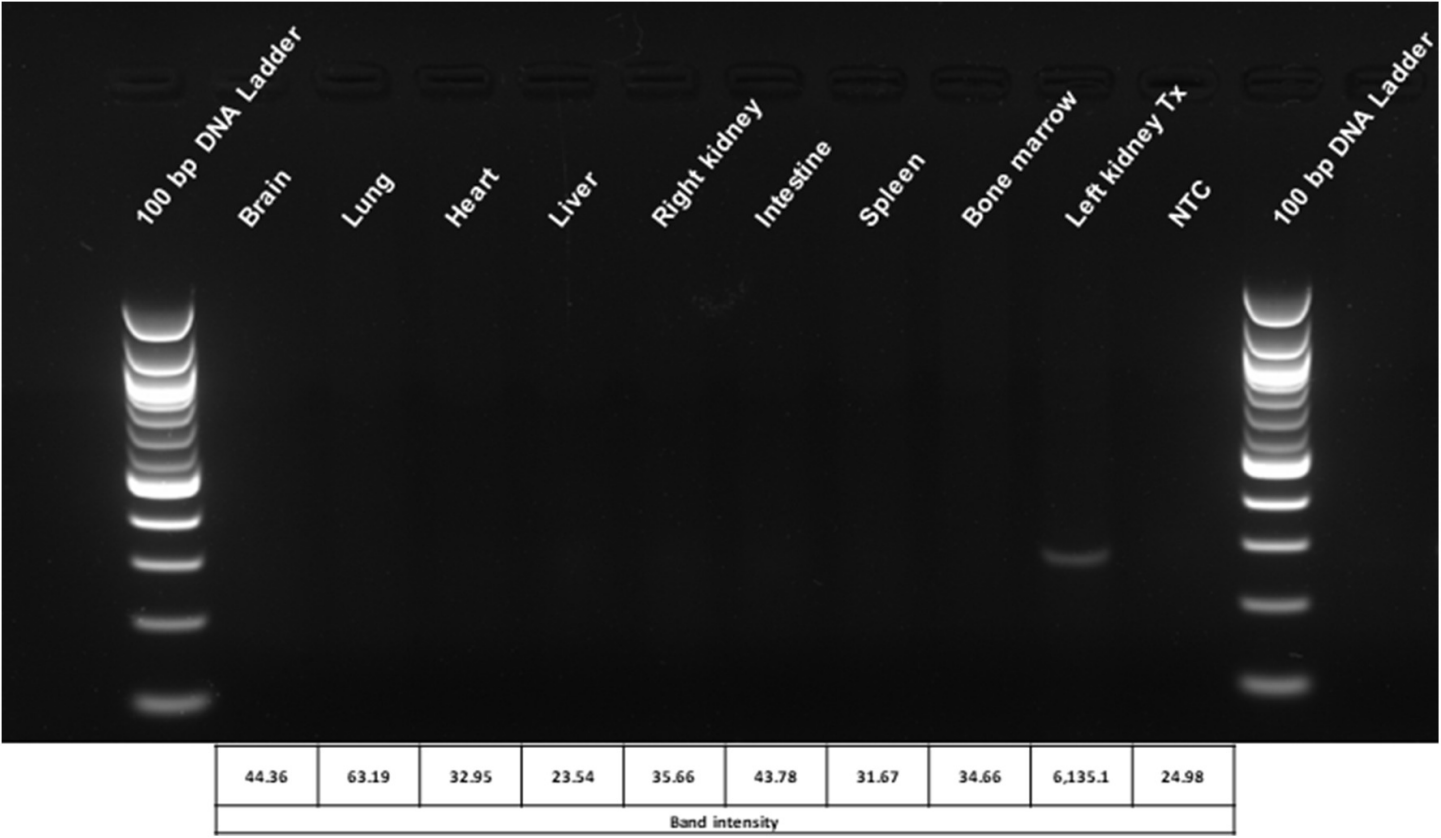
Biodistribution analysis of the lentiviral vector used for genetic modification of the kidneys during *ex vivo* perfusion. The localization of the vector is restricted to the engineered graft. Representative picture of the lentiviral vector biodistribution assay in rat organs and tissues 6 weeks after transplantation with *ex vivo* genetically engineered kidneys. Densitometric analysis of the bands was performed using the BioRad Image Lab 6.0.1 software. Tx, genetically engineered transplanted kidney; NTC, no template control.

*Ex vivo* organ perfusion permits the precise genetic engineering of the target organ, strongly reducing the risk for undesired off-target effects or adverse reactions due to not modifying the cells or other organs. Hence, after transplantation of genetic engineered kidneys, we have assessed different organs for the presence of the lentiviral vector. The vector could not be found in any other organ and was exclusively restricted to the modified renal graft (Figure 7).

## Discussion

In this study, we have demonstrated that the kidney can be genetically engineered in a permanent manner toward reduction of its immunogenicity.

### Organ Gene Therapy

Recently, gene therapeutic approaches have demonstrated to be successful in the treatment of several diseases such as inherited retinal dystrophies, cancer, hemoglobinopathies and neuromuscular diseases using non-viral or viral-based vector technologies (26–30). Furthermore, several pre-clinical studies show significant progresses in the development of gene therapeutic strategies at organs such as the lung and the liver (31, 32). Selection of the method to deliver of the therapeutic vectors remains an essential and crucial hallmark; on the one hand to achieve organ specificity and high transduction efficiencies and on the other hand to ensure maintenance of organ quality during the genetic engineering process. Despite many efforts, *in vivo* delivery of gene therapeutic vectors has been proven to be inefficient and unspecific (17).

### *Ex vivo* Organ Perfusion in Organ Engineering

*Ex vivo* normothermic perfusion has been extensively studied during the past decade and it has allowed to monitor function and circulation in marginal organs, such as in case of donation after circulatory death or from extended criteria donors, in kidney, lung, heart and liver transplantation studies and also to deliver certain therapeutic strategies (33–35). During *ex vivo* normothermic perfusion the donated organ undergoes machine perfusion with warm and oxygenated blood or preservation solution prior to transplantation. The feasibility of *ex vivo* normothermic perfusion for long periods such as 24 h was previously demonstrated (36). The prolonged warm perfusion time with maintenance of functionality creates a window of opportunity for various therapeutic interventions. Among those, genetic organ engineering is one of the most promising opportunities to correct monogenetic diseases or support allograft survival after transplantation.

### Immuno-Engineering of the Kidney

Recently, we have shown the possibility to engineer the lung endothelium by lentiviral vectors during *ex vivo* normothermic perfusion in a porcine model (18). In a porcine heart study it has been reported that the intravascular delivery of adenoviral (Ad) vectors encoding the luciferase gene led to widespread transgene expression in the allograft (37). The intrabronchial route was also exploited in porcine lungs for genetic modification with human IL-10 encoding Ad vector during *ex vivo* normothermic perfusion (38). Furthermore, it has been reported that glomeruli had been extensively transduced with an Ad vector encoding for β-galactosidase after normothermic EVKP in a porcine model (39). These studies indicate that EVKP creates favorable conditions for genetic organ engineering. In contrast to Ad or Ad-associated vectors, lentiviral vectors enable a permanent transgene expression which might be essential to support long-term graft survival. Human Leukocyte Antigen (HLA) mismatches between donor and recipients remain a major obstacle in allogeneic transplantation. However, ~38% of kidney graft failure is expected to be triggered by non-HLA-dependent factors. In fact, evidences for the relevance of non-HLA antibodies in leading to kidney transplant dysfunction is increasing. Tissue injury caused by ischemia-reperfusion or vascular injury may favor the upregulation of cryptic autoantigens on the graft endothelial cells such as the angiotensin II type 1 receptor and serve as a target for autoantibodies after transplantation (40, 41). Recently, RNAi has gained plenty of attention in organ transplantation in particular to prevent ischemia reperfusion injury (IRI) by silencing the expression of different genes such as Caspase 3, IKKβ, Fas or RelB. Most of these studies used chemically modified siRNAs or siRNA sequences encoded by plasmid DNA administrated via arterial or venous infusion or injection to prevent IRI. Gene silencing effects using stabilized siRNAs were detectable by periods of 2 to 3 weeks (42–46). In this study, we have selected RNAi as technology to silence MHC class I and II expression and lentiviral vectors to ensure a prolonged expression of the shRNAs. It is well-known that the abrogation of MHC class I expression triggers NK cell cytotoxicity. In previous studies, we have demonstrated that the residual expression of MHC class I molecules is required to prevent NK cell cytotoxicity (22). Thus, we have selected RNAi as the gene regulatory strategy to downregulate MHC expression and not gene editing tools such as CRISPR/Cas9 or TALENs which would generate a complete MHC knockout. In addition, gene regulatory strategies also enable the re-expression of the targeted gene in case of interest by using Tet-ON/OFF promoters. This may be beneficial in case of infections or tumor development. Here, we showed the lentiviral-mediated transduction of the kidney during *ex vivo* perfusion and the sustained transgene expression after ktx as demonstrated by the levels of luminescence in plasma and urine samples of the animals 6 weeks after transplantation (Figure 2, Supplementary Figure 2). This stable transduction of the kidney grafts is in line with our results in the porcine lung model showing the lentiviral delivery of shRNAs to induce a specific downregulation of MHC class I and MHC class II transcripts (18). We have detected an increase in LDH during perfusion time, however this tendency has also been previously observed in different studies focused in the *ex vivo* recirculating perfusion of organs such as the kidney, liver and lung. This was mainly explained by periods of warm ischemia prior perfusion and by the effect of using pumps and cardiopulmonary bypass during perfusion (18, 47–49). Importantly, we showed that the transduction of the kidney with lentiviral vectors did not cause additional tissue injury or cell death as detected by the LDH levels in the kidney perfusate and renal tissue histological analysis. In order to minimize the risks for cell damage during perfusion, the perfusion solution was oxygenated in our system. Previous studies have indicated the benefits of oxygenating the perfusion solution already during hypothermic perfusion by reducing oxidative stress and supporting the energy status in presence of low metabolic rates (50, 51). During sub-normothermic to normothermic perfusion the increased metabolism demands an appropriate oxygen supply, but recent studies indicate that normothermic perfusion with reduced perfusate oxygenation for a limited period of time may also be possible without severely compromising renal function or tissue integrity (52).

Cytokines play essential roles in the maintenance of tissue homeostasis and host defense. However, dysregulation of typical cytokine release patterns may trigger detrimental immune cascades after transplantation (25). Previous studies reported that the procedure of organ *ex vivo* perfusion itself elicits an inflammatory reaction with increasing cytokine levels after harvest and placement of organs on the pump-driven perfusion system (53). In this study, we compared cytokine signatures between rat kidneys perfused without and with lentiviral vectors. An increase in the secretion of MIP-1α, MIP-2, IP-10, IL-10, and EGF was detected in the perfusate of organs exposed to lentiviral vectors during perfusion in this model. In contrast, the secretion of cytokines IL-12p70, IL-17, MCP-1, and IFN-γ was lower in genetically engineered organs. The impact of this change in the cytokine secretion pattern in the transplantation outcome needs to be investigated in detail in future experiments. Currently, different approaches to prevent the release of pro-inflammatory cytokines by *ex vivo* perfused organs based on the use of membranes or small molecules are being developed to avoid tissue impairment during perfusion or the activation and polarization of immune responses after transplantation (54, 55).

### Safety

Lentiviral vectors are powerful tools for genetic engineering and their use in clinical trials is rising. Nevertheless, the use of lentiviral vectors might be associated with safety concerns such as an increased risk for tumorigenesis (56). In contrast to the *in vivo* application of lentiviral vectors, *ex vivo* organ perfusion allows for the selective genetic modification of the target organ thereby reducing the possibility for off-target and systemic adverse effects. In the transplantation setting an *ex vivo* period of the allograft between transplant procurement and recipient transplantation is inevitable. This inevitable *ex vivo* period of the graft provides a unique opportunity for an *ex vivo* transplant engineering taking advantage of not having to accept systemic off-target effects. In our study, we showed that after transplantation of transduced kidneys the integrated vector DNA was exclusively restricted to the genetically engineered organs. Hence, delivery of the lentiviral vector during *ex vivo* perfusion not only permits the efficient transduction of the organ and stable transgene expression, but simultaneously supports the safety of this procedure.

## Conclusions

In this study, we have demonstrated the feasibility to engineer the kidney during *ex vivo* perfusion over prolonged time periods. Furthermore, levels of MHC class I and II transcripts were also stably downregulated. In future studies, the benefit of invisible of MHC-silenced allografts will need to be investigated toward improvement of allograft survival, function, and reduction of development of donor specific antibodies. Long term goals would be to reduce the amount of immunosuppression and ideally to induce tolerance toward the allograft.

## Data Availability Statement

All datasets generated for this study are included in the article/Supplementary Material.

## Ethics Statement

The animal study was reviewed and approved by Nidersächsisches Landesamt für Verbraucherschutz und Lebensmittelsicherheit (AZ: 17/2476).

## Author Contributions

CF and RB designed the study. YY, EV, FH, TR, and NW performed experiments. FG and SR performed transplantation experiments. JS, JB, and FG performed histological analyses. DW contributed to the animal experiments. CM contributed with critical advice and to construct the miniature organ perfusion system. CF, RB, FG, NW, YY, and EV analyzed the data and wrote the manuscript.

### Conflict of Interest

The authors declare that the research was conducted in the absence of any commercial or financial relationships that could be construed as a potential conflict of interest.
