# Supplementary material for: Genetic Engineering of the Kidney to Permanently Silence MHC Transcripts During ex vivo Organ Perfusion
Source: Front Immunol. 2020 Feb 19;11:265. doi: 10.3389/fimmu.2020.00265 (PMC7042208; doi:10.3389/fimmu.2020.00265)
Supplement: Supplementary file 1 [file Data_Sheet_1.pdf]

Perfusion system design

The rat kidney perfusion system consisted of a roller-pump (Reglo ICC, Ismatech, Cole-Parmer, Wertheim, Germany), a circulating water bath (MA-4, Julabo, Seelbach, Germany) combined with a glass heat-exchanger (Central Research Devices Service Unit, Hannover Medical School, Hannover, Germany) and an oxygenator. The latter was built of 3 m silicone tubing with ID 1.5 mm and OD 2.3 mm (Deutsch & Neumann, Berlin, Germany) placed in a Büchner flask supplied with carbogen (Linde, Guildford, UK). To monitor the perfusion parameters, an oxygen sensor in a form of a flow-through cell with an oxygen transmitter (PreSens, Regensburg, Germany), as well as temperature, pressure and flow sensors (all PendoTECH, Princeton, USA) were integrated. The temperature, pressure and flow sensors were connected to PressureMAT Sensor Monitor sending the readings to a PC with PressureMAT-Plus data acquisition software (both from PendoTECH, Princeton, USA).

Kidney transplantation in rats

After induction of anesthesia in an induction chamber with 4% isoflurane (CP-Pharma, Burgdorf, Germany) the rats were placed on a heating pad in a supine position and anesthesia was continued via a nose mask with 2% isoflurane for maintenance at room air. For analgesia carprofen 5mg/kg was injected subcutaneously (sc) prior to start of the surgical procedure and also 200mg/kg metamizol was given at the end of the procedure sc. In addition, the rats received metabmizol 200mg/kg via drinking water during the next three days. For the donor operation median laparotomy was performed exposing the retroperitoneum. After removal of the Gerota's fascia and perirenal fat, the aorta, vena cava, renal artery, vein and ureter of the left kidney were identified. The aorta and vena cava proximal were ligated. The aorta distal was cannulated with a 20-gauge, 1.1 x 33 mm Vasofix Safety Braunüle (B.Braun, Melsungen,

Germany) and the kidney was flushed with 20 ml of 4°C cold Custodiol (Dr. Franz Köhler Chemie, Bensheim, Germany) *in situ.* The ureter was excised close to the bladder and the kidney with the renal vessels and the ureter was retrieved and placed immediately on ice.The left native kidney of the recipient was retrieved as described above and the vessels and the ureter were closed by tight sutures. For the transplantation of the donor kidney an end-to-side anastomosis of the aorta was performed first, followed by the renal vein to vena cava anastomosis with continuous stitches using Ethilon monofil, USP 8/0, 15cm, BV-2 suture material (Ethicon, Norderstedt, Germany). The ureter was anastomosed to the bladder dome. After allowing reperfusion by removing the vascular clamps perfusion was visually controlled. The abdomen was closed using a non-absorbable suture material on the rectus muscle with continuous suture. The outer skin was closed in an interrupted manner with a non-absorbable thread. After surgery the animals were placed for recovery in a clean cage with access to food and water *ad libitum* on a heating pad and closely monitored for at least 2 hours prior return to the housing facility. Animal well-being was monitored daily throughout the observation period of 6 weeks. Normal renal function was maintained by the right native kidney of the recipient in all groups.

Supplementary figure 1. Representative graph of the kidney perfusion parameters controlled in the course of lentiviral transduction of the rat kidney during ex vivo perfusion. The graph depicts pressure, flow, temperature, oxygen saturation and pump speed.

Supplementary figure 2. Levels of bioluminescence in the supernatants of kidney cells.

Increased secreted NanoLuc Luciferase activity was detected in cell culture supernatants of CD31+ endothelial cell-enriched kidney cell populations in comparison to CD31+ cells-

depleted cell cultures. Kidney cells were isolated from a genetically engineered kidney that was transduced with the NanoLuc reporter gene encoding vector during ex vivo perfusion. After collagenase and dispase digest, the cell suspension was labeled with an antibody against CD31 and sorted via magnetic-activated cell sorting. The isolated CD31+ cell-enriched and CD31+ cell-depleted populations were cultured in endothelium growth medium. NanoLuc activity was measured in cell culture supernatants on day 4 and normalized to cell number in both populations. Cells isolated from a non-genetically engineered kidney were used as control. RLU - relative luminescence units.
