# Supplementary figures and images for: Genetic Engineering of the Kidney to Permanently Silence MHC Transcripts During ex vivo Organ Perfusion
Source: Front Immunol. 2020 Feb 19;11:265. doi: 10.3389/fimmu.2020.00265 (PMC7042208; doi:10.3389/fimmu.2020.00265)

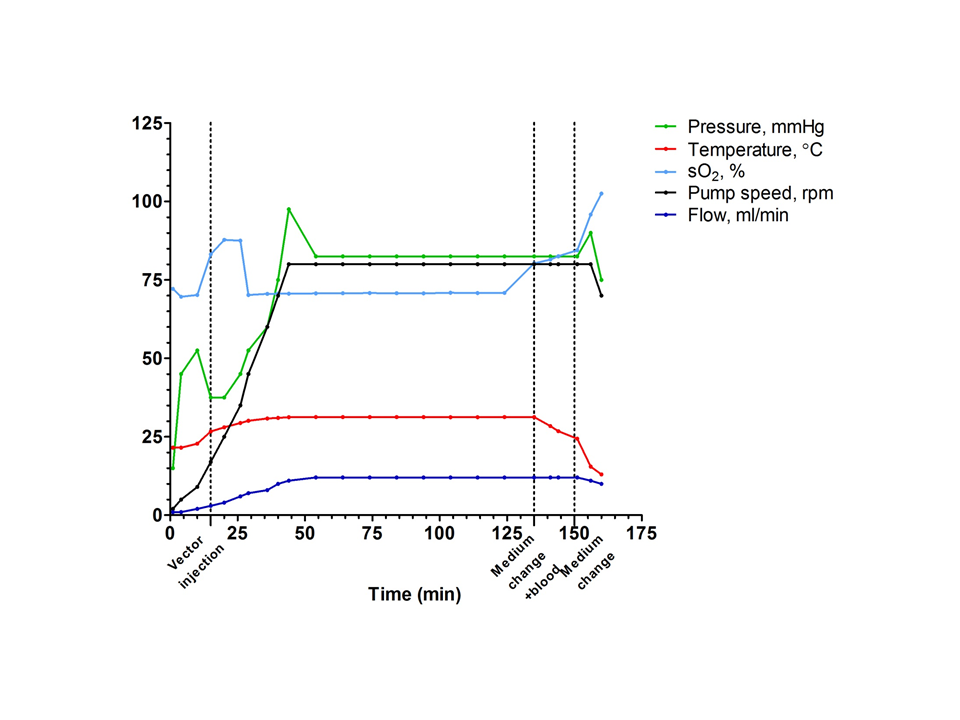

Supplement: Supplementary file 2 [file Image_1.TIF]

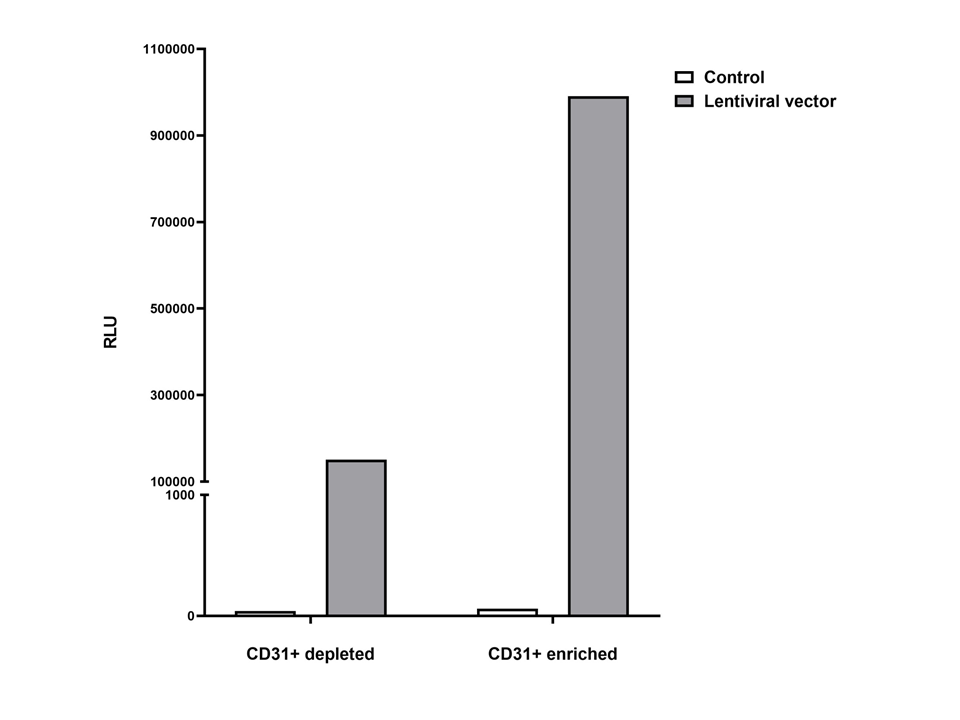

Supplement: Supplementary file 3 [file Image_2.TIF]
